# Comparative Proteomic Analysis of *Dipsacus asperoides* Roots from Different Habitats in China

**DOI:** 10.3390/molecules25163605

**Published:** 2020-08-08

**Authors:** Haijun Jin, Hua Yu, Haixia Wang, Jia Zhang

**Affiliations:** Resource Institute for Chinese & Ethnic Materia Medica, Guizhou University of Traditional Chinese Medicine, Guiyang 550025, China; wangnajhjxm@163.com (H.Y.); haixiaxm@163.com (H.W.); anyone2394@126.com (J.Z.)

**Keywords:** *Dipsacus asperoides*, iTRAQ, proteomic

## Abstract

*Dipsacus asperoides* is a kind of Chinese herbal medicine with beneficial health properties. To date, the quality of *D. asperoides* from different habitats has shown significant differences. However, the molecular differences in *D. asperoides* from different habitats are still unknown. The aim of this study was to investigate the differences in protein levels of *D. asperoides* from different habitats. Isobaric tags for relative and absolute quantification (iTRAQ) and 2DLC/MS/MS were used to detect statistically significant changes in *D. asperoides* from different habitats. Through proteomic analysis, a total of 2149 proteins were identified, of which 42 important differentially expressed proteins were screened. Through in-depth analysis of differential proteins, the protein metabolism energy and carbohydrate metabolism of *D. asperoides* from Hubei Province were strong, but their antioxidant capacity was weak. We found that three proteins, UTP-glucose-1-phosphate uridylyltransferase, allene oxide cyclase, and isopentyl diphosphate isomerase 2, may be the key proteins involved in dipsacus saponin VI synthesis. Eight proteins were found in *D. asperoides* in response to environmental stress from different habitats. Quantitative real-time PCR analysis confirmed the accuracy and authenticity of the proteomic analysis. The results of this study may provide the basic information for exploring the cause of differences in secondary metabolites in different habitats of *D. asperoides* and the protein mechanism governing differences in quality.

## 1. Introduction

*Dipsacus asperoides* is a traditional Chinese medicinal plant. The root of this plant is generally used as a medicine and is frequently prescribed by Chinese doctors for the treatment of back pain, limb paralysis, flutter trauma, tendon injuries, and fractures [1]. *D. asperoides* is widely distributed in southwestern China. In recent years, because the domestic and foreign demand for the herb has been increasing annually, the price of *D. asperoides* has continued to rise. Therefore, the wild *D. asperoides* had been harvested excessively, and the wild population of this herb has decreased [1]. Some large-scale cultivation bases for *D. asperoides* were established in Hefeng city, Hubei province (29°82′58″ N, 110°58′22″ E, H group); Xichang city, Sichuan province (28°4′4″ N, 102°8′36″ E, S group); Xifeng city, Guizhou province (27°10′73″ N, 106°74′38″ E, G group); and Jianchuan city, Yunnan province (26°31′91″ N, 99°85′24″ E, Y group), China. Hefeng city of Hubei province is the main producing area of *D. asperoides*, and the other three areas are emerging producing areas.

However, due to the different growth environments, the accumulated active components and the quality of *D. asperoides* have shown marked differences in different cultivation bases [2,3,4]. At present, research on *D. asperoides* has primarily focused on chemical composition [5], cultivation [6], pharmacology [7], and transcriptomics [1]. Triterpenoid saponins, such as dipsacus saponin VI, macranthoidin A and HN saponin F, are the principal active ingredients of *D. asperoides* [8]. Dipsacus saponin VI is an important quality indicator of *D. asperoides* and has also been written into the Chinese Pharmacopoeia [9]. Current reports on the differences in *D. asperoides* quality in different habitats are mostly concentrated on the difference in the content of dipsacus saponin VI. There are no reports on the molecular mechanisms related to *D. asperoides* quality differences in different habitats. To explore the causes of differences in secondary metabolites in different habitats of *D. asperoides* and the mechanisms that underlie its quality, it is necessary to study the proteomics of this herb.

Isobaric tags for relative and absolute quantification (iTRAQ) is one of the most important techniques that quantifies proteins on the basis of peptide labeling and allows the identification and accurate quantification of proteins from multiple differential samples within broad dynamic ranges of protein abundance [10,11]. Therefore, it has been used to reveal differentially expressed proteins in cells and organelles under various physiological or environmental conditions [12,13,14]. There have also been some studies on traditional Chinese herbs using iTRAQ [15], such as *Dendrobium officinale* [16], *Pseudostellaria heterophylla* [17] and *Gastrodia elata blume* [18].

In the present work, we found the dipsacus saponin VI content in *D. asperoides* from different habitats was significantly different, and the samples from Hubei contained the highest dipsacus saponin VI. Then a comparative proteomics technology was used to study *D. asperoides* from different habitats to identify the differentially expressed proteins between different habitats. In addition, quantitative real-time PCR (qRT-PCR) assays were used to validate the proteomic data. Bioinformatic analyses indicated that protein metabolism energy and carbohydrate metabolism of *D. asperoides* from Hubei Province were strong, but their antioxidant capacity was weak. We also found that three proteins, UTP-glucose-1-phosphate uridylyltransferase, allene oxide cyclase, and isopentyl diphosphate isomerase 2, may be the key proteins involved in dipsacus saponin VI synthesis. Our findings of candidate enzymes involved in dipsacus saponin VI biosynthes provides novel information for us to understand the synthesis pathway of triterpenoid saponins in the future. The results of this study may provide the basic information for exploring the cause of differences in secondary metabolites in different habitats of *D. asperoides* and the protein mechanism governing differences in quality.

## 2. Results

### 2.1. Content of Dipsacus Saponin VI from Different Habitats

**Figure 1:**
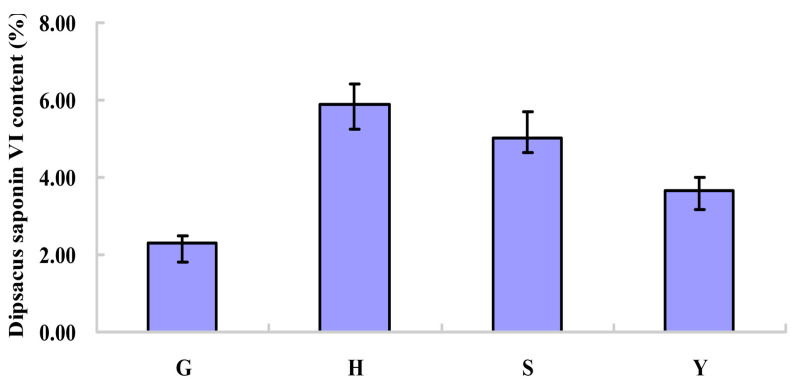
Contents of dipsacus saponin VI in *D. asperoides* of different habitats. G, H, S, and Y represent the dipsacus saponin VI content in *D. asperoides* from Guizhou, Hubei, Sichuan, and Yunnan, respectively. The % in the ordinate refers to the percentage of dry weight.

From Figure 1, the dipsacus saponin VI content in *D. asperoides* from different habitats was significantly different (*p* < 0.05), and the samples from Hubei contained the highest dipsacus saponin VI.

### 2.2. Protein Identification

**Figure 2:**
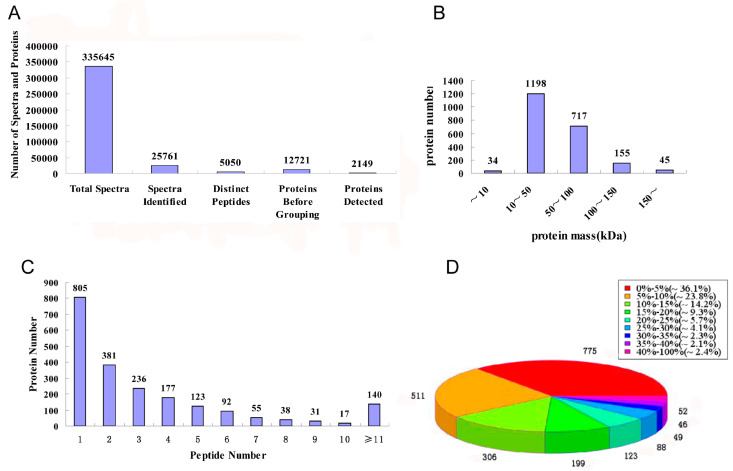
Identification and analysis of the *Dipsacus asperoides* proteome. (**A**) Total spectra, spectra identified, distinct peptides, proteins before grouping, and proteins detected from iTRAQ proteomic analysis. (**B**) Identified proteins were grouped based on their protein mass. (**C**) Number of peptides that match to proteins as shown by Protein Pilot 5.0. (**D**) According to the protein sequence coverage, the identified proteins were classified into pie charts.

Through iTRAQ-LC-MS/MS, a total of 335,645 spectra, 25,761 spectra identified, 5050 distinct peptides, 12,721 proteins before grouping, and 2149 proteins (Table S1) were acquired (Figure 2A). There were 34, 1198, 717, 155, and 45 proteins with molecular weight less than 10 kDa, 10–50 kDa, 50–100 kDa, 100–150 kDa, and more than 150 kDa, respectively (Figure 2B). The distribution of peptide numbers is shown in Figure 2C. The proteins with a single peptide, 2–5 peptides, 6–10 peptides, and above 11 peptides consisted of 805, 917, 233, and 140, respectively. Protein sequence coverage with 40–100%, 30–40%, 20–30%, 10–20%, and under 10% variation accounted for 2.4%, 4.4%, 9.8%, 23.5%, and 59.9%, respectively (Figure 2D).

### 2.3. Bioinformatics Analysis of Differential Proteins

**Figure 3:**
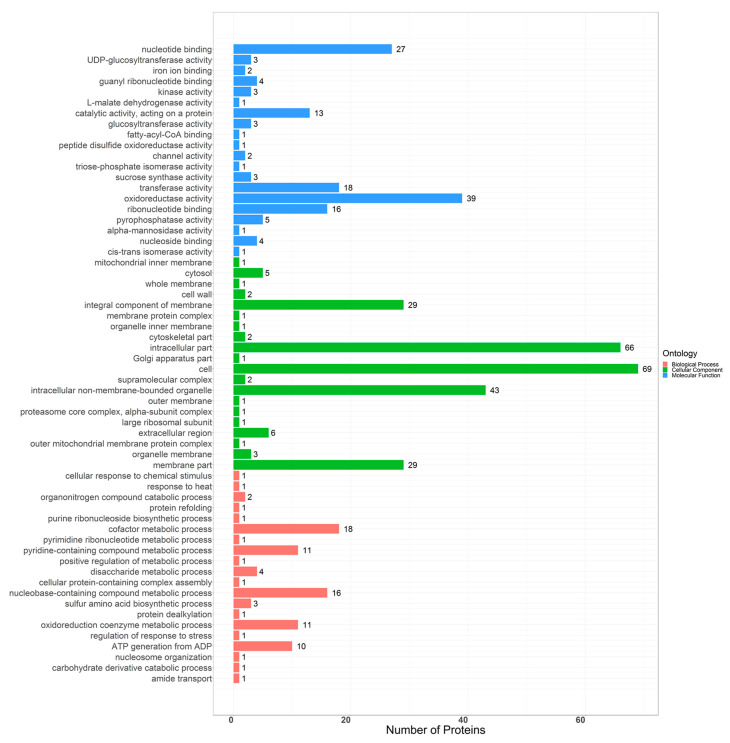
Gene ontology of differential proteins.

**Figure 4:**
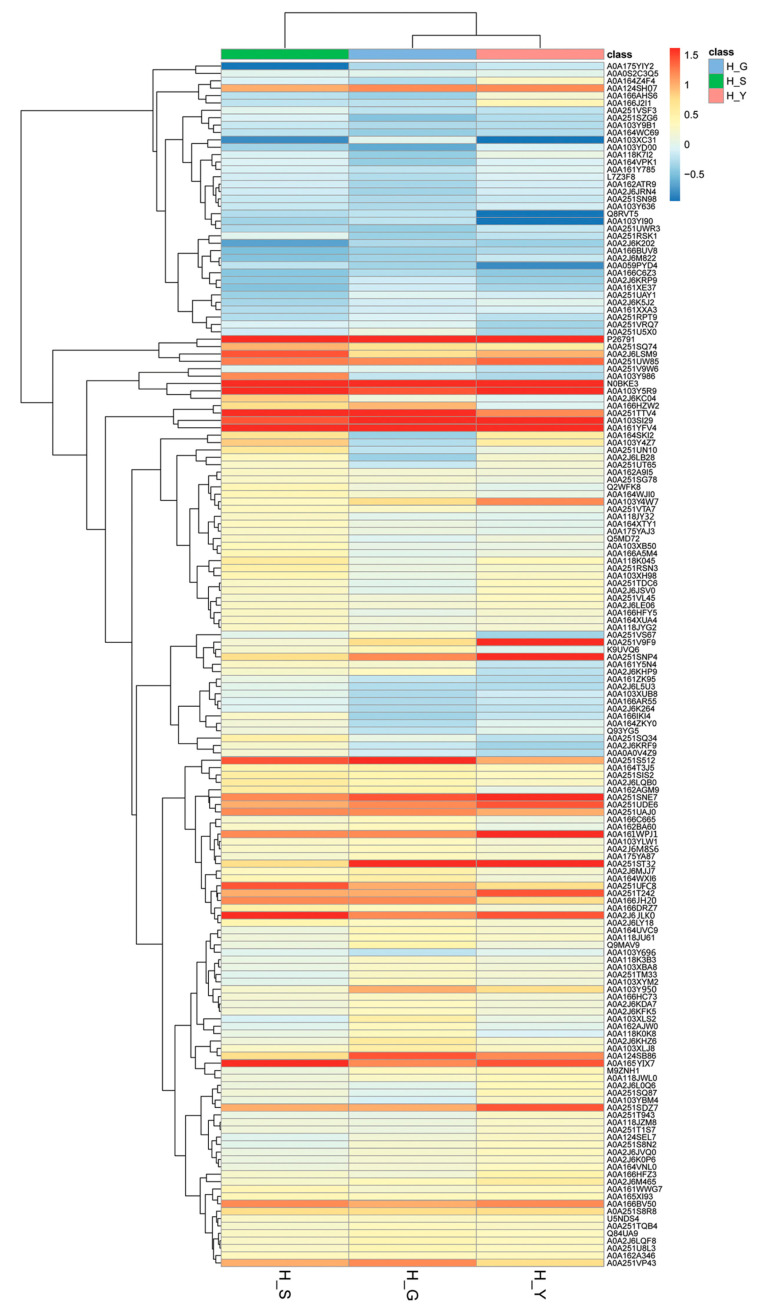
Thermal maps of differences in proteins expression. One grid in a thermal map stands for one protein, and the color for the magnitude. A deeper color means larger magnitude, while red and blue mean the protein expression is upregulated and downregulated, respectively. The left and right sides represent the H vs. S, H vs. G, and H vs. Y groups, respectively. One row means the expression level of each protein in a group, while the column means the differences in proteins expression.

By ANOVA analysis, the protein differential significance was *p* < 0.05, and the difference fold was greater than 1.2 as the differential protein. In the comparison between the H group and the other three groups (Y, S, G group), we screened a total of 202 differentially expressed proteins (Table S2). The 202 differential proteins were sent into gene ontology (Figure 3). These proteins were classified by cellular component and found to be mainly involved in cell, intracellular part, intracellular non-membrane-bounded organelle, integral component of membrane, and membrane part. These proteins were classified by biological process mainly into cofactor metabolic process, nucleobase-containing compound metabolic process, pyridine-containing compound metabolic process, oxidoreduction coenzyme metabolic process, and ATP generation from ADP. Their molecular functions were mainly divided into oxidoreductase activity, nucleotide binding, transferase activity, ribonucelotide binding, and catalytic activity. In addition, we also mapped heat maps for different proteins between different groups (Figure 4). We could see from Figure 4 that the differential expression levels of differential proteins between different groups were different.

As shown in Table 1, these 202 differentially expressed proteins were aligned to the KEGG database, and they were found to be primarily involved in ribosome (15.84%), carbon metabolism (8.91%), biosynthesis of amino acids (5.94%), carbon fixation in photosynthetic organisms (4.95%), starch and sucrose metabolism (4.95%), glycolysis/gluconeogenesis (4.46%), phenylpropanoid biosynthesis (4.46%), cysteine and methionine metabolism (2.97%), MAPK signaling pathway-plant (2.48%), glyoxylate and dicarboxylate metabolism (1.98%), cyanoamino acid metabolism (1.98%), glutathione metabolism (1.98%), ubiquitin mediated proteolysis (1.49%), protein processing in endoplasmic reticulum (1.49%), plant-pathogen interaction (1.49%).

**Figure 5:**
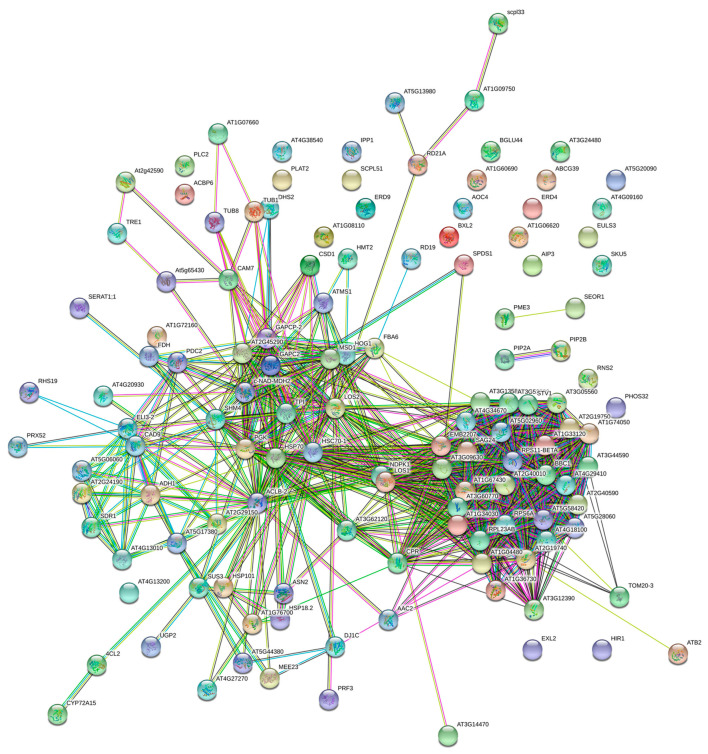
Interaction network analysis of differential expression of proteins. In this network, nodes are proteins, lines represent functional associations between proteins, and the thickness of the lines represents the level of confidence in association reported.

To find out if there is a functional link between the different proteins, we performed a STRING grid analysis of the differential proteins (Figure 5). It was found that 128 of the 202 differential proteins were aligned to the STRING database of Arabidopsis homology (Table S3). Interactions in proteins that participated in heat shock proteins, ribosomal proteins, and energy metabolism related proteins had a high level of coexpression. The heat shock proteins were HSP101, HSP70, HSC70-1, HSP18.2. 40S ribosomal protein (AT2G40590, AT3G52580, AT3G60770, AT4G34670, AT5G02960, AT5G28060, AT5G58420, AT1G34030, AT2G19750, RPS11-BETA, RPS6A) and 60S ribosomal protein (AT2G40010, AT3G05560, AT3G09630, AT3G13580, AT1G33120, AT1G67430, AT1G74050, AT2G19740, AT4G18100, AT1G04480, AT4G29410, EBM2207, SAG24, RPL23AB) were involved in ribosomal proteins. Glyceraldehyde 3-phosphate dehydrogenase (GAPC2 and GAPCP-2), bifunctional enolase 2/transcriptional activator (LOS2), transketolase (AT2G45290), triosephosphate isomerase (TPI), adenosylhomocysteinase 1 (HOG1), methionine synthesis 1(ATMS1), malate dehydrogenase (c-NAD-MDH2), ATP citrate lyase subunit B 2 (ACLB-2), and pyruvate decarboxylase-2 (PDC2) participated in energy metabolism-related proteins.

Through the integrated analysis of GO/KEGG/STRING of 202 differentially expressed proteins, we focused on screening 42 significantly differentially expressed proteins and classifying them into 7 categories: energy and carbohydrate metabolism, protein metabolism, amino metabolism, stress and defense, nucleic acid metabolism, cell wall synthesis, and secondary metabolism (Table 2).

### 2.4. Verification of Transcriptional Expression of Candidate Genes for The Differential Expression of Proteins

**Figure 6:**
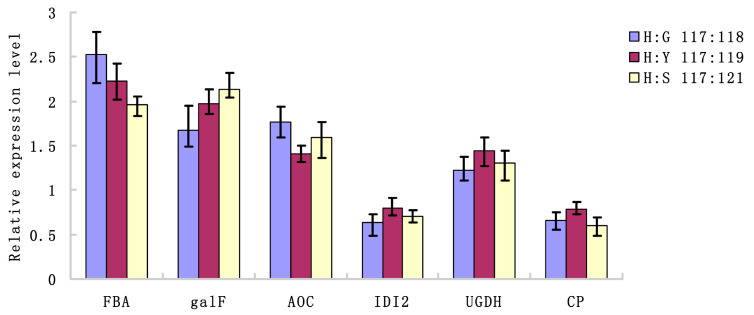
Relative expression abundance analysis of differential proteins at the transcriptional level by RT-qPCR. The *18sRNA* gene was used as the internal control. To determine the relative fold differences for each gene, the Ct value of the genes was normalized to the Ct value for the 18sRNA (control gene), and the relative expression was calculated relative to a calibrator using the formula 2^−ΔΔ*C*t^. All the values shown are means ± SE.

To verify the expression of differential proteins at the transcription level, we analyzed six candidate differential expression of proteins consisting of fructose-bisphosphate aldolase (*FAB*), UTP-glucose-1-phosphate uridylyltransferase (*galF*), allene oxide cyclase (*AOC*), isopentyl diphosphate isomerase 2 (*IDI2*), UDP-glucose 6-dehydrogenase (*UGDH*), and carboxypeptidase (*CP*). From the RT-qPCR analysis (Figure 6), the transcription levels of these genes were correlated with the expression of their respective proteins, further validating our comparative proteomic study.

## 3. Discussion

The genetic material in the nucleus of Chinese medicinal materials from different producing areas may not change, but the differences in eco-environmental factors of different producing areas may lead to differences in the expression of related proteins in Chinese herbal medicines [17]. Therefore, the use of proteomics technology is conducive to revealing the reasons the differences in the quality of Chinese herbal medicines in different habitats at the molecular level. By analyzing the GO, KEGG, and STRING grids of differentially expressed proteins in *D. asperoides* roots from different producing areas, we divided them into the following 7 categories.

### 3.1. Energy and Carbohydrate Metabolism

**Figure 7:**
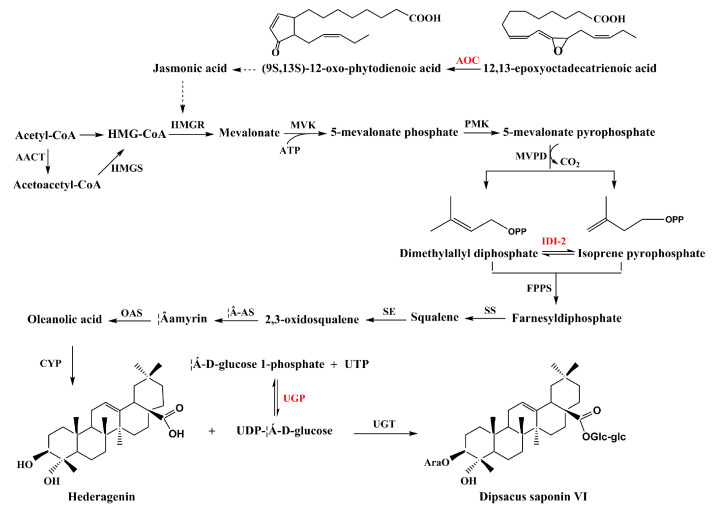
Putative dipsacus saponin VI biosynthesis pathway in cell. The enzymes found in this study are shown in red font. AACT, acetoacetyl-CoA thiolase; HMGS: HMG-CoA synthetase; HMGR, HMG-CoA reductase; MVK, mevalonate kinase; PMK, phosphomevalonate kinase; MVPD, 5-mevalonate pyrophosphate decarboxylase; IDI-2, isopentyl diphosphate isomerase 2; FPPS, farnesyl diphosphate synthase; SS, squalene synthase; SE, squalene epoxidase; β-AS, β- amyrin synthase; OAS, oleanolic acid synthase; CYP, Cytochrome P450 proteins; UGP, UTP-glucose-1-phosphate uridylyltransferase; UTP, Uridine triphosphate; UDP, Uridine diphosphate; UGT, Uridine diphosphate glucuronic acid transferase; AOC, allene oxide cyclase.

By comparing the H group with the Y, S, and G groups, we screened 12 differentially expressed proteins involved in energy and carbon metabolism, 10 of which were upregulated and 2 of which were downregulated in the H group. The main up-regulated proteins were fructose-bisphosphate aldolase (A0A161YFV4), UTP-glucose-1-phosphate uridylyltransferase (A0A103Y5R9), and glyceraldehyde-3-phosphate dehydrogenase (N0BKE3). Fructose-bisphosphate aldolase (EC 4.1.2.13, FBA) is an enzyme catalyzing a reversible reaction that decomposes aldolfructose 1,6-bisphosphate into the glyceraldehyde 3-phosphate (G3P) and triose phosphates dihydroxyacetone phosphate (DHAP). FBA genes have been shown to be involved in many important biofunctions, e.g., FBA plays a vital role in plant development, signal transduction, and abiotic stress response [19]. As a key enzyme of carbohydrate metabolism and cell wall biosynthesis, UTP-glucose-1-phosphate uridylyltransferase (EC 2.7.7.9, UGP) can catalyze the reversible reaction between glucose-1-phosphate and UDP-glucose (UDP-Glc) [20]. UDP-Glc, as a glycosyl donor in cells, can be involved in the glycosylation of many molecules in cells. Since dipsacus saponin VI is the primary active ingredient in *D. asperoides*, there must be a glycosylase catalyzing the glycosylation of substituted hydroxyl groups of hederagenin [1]. We speculate that UDP glucose catalyzed by UTP-glucose-1-phosphate uridylyltransferase, as a glycosyl donor, participates in the important glycosylation of hederagenin to dipsacus saponin VI (Figure 7). Glyceraldehyde-3-phosphate dehydrogenase (EC 1.2.1.12, GAPDH) can catalyze the conversion of glyceraldehyde-3-phosphate (G3P) to 1,3-biphosphoglycerate in the presence of NAD+ and inorganic phosphate, which is a key enzyme in the glycolytic pathway [21]. Non-glycolytic functions in DNA repair, signal transduction cascades, apoptosis, and transcriptional regulation of plant GAPDH have been demonstrated as well, especially in abiotic stress responses [22]. The number of upregulated proteins is considerably higher than the number of downregulated proteins; therefore, we speculate that energy and carbon metabolism in Hubei *D. asperoides* were stronger than those in Sichuan, Guizhou, and Yunnan.

### 3.2. Protein Metabolism

According to our proteomic profiles, 16 differentially expressed proteins were involved in the cellular protein metabolism process, including 12 proteins with upregulated expression and 4 proteins with downregulated expression in H group. The main up-regulated proteins were putative eukaryotic aspartyl protease family protein (A0A251TTV4), heat shock protein 70 (P26791), putative heat shock protein 81-2 (A0A251ST32), and elongation factor 1-alpha (A0A2J6JLK0). Putative eukaryotic aspartyl protease family protein may be involved in the specific degradation of polypeptides during the defense reactions [23]. Recent studies have indicated that aspartyl protease could trigger autophagy and plant defense, providing a critical link between fungal identification and the induction of resistance and cell death [24]. Elongation factor 1-alpha catalyzes the binding of aminoacyl-tRNA to the ribosome A-site through a GTP-dependent mechanism, which is an important component of protein biosynthesis [25]. Several studies have shown that the expression of eEF1A genes may vary during low temperature, developmental stages, high temperature, drought, low oxygen, light, chemical induction (e.g., ethephon), physical wounding, and pathogen attack [26]. Heat shock protein 70 prevents the aggregation and promotes the refolding of misfolded denatured proteins, solubilizes aggregated proteins, and cooperates with cellular degradation machineries to clear aberrant proteins and protein aggregates. Thus, Hsp70s act as sentinel chaperones, guarding cells from the deleterious effects of a wide range of proteotoxic stresses, pathophysiological conditions, and organismal aging that cause protein homeostasis imbalance [27]. Putative heat shock protein 81-2 has a similar effect to that of heat shock protein 70, and also protects cells against injuries associated with various stressors [28]. Thus, we speculate that in terms of heat shock proteins, the ability to respond to abiotic stress in Hubei *D. asperoides* were stronger than those in Sichuan, Guizhou, and Yunnan. Other upregulated proteins were also primarily related to the anabolism of intracellular proteins. The down-regulated proteins are primarily involved in the biological processes of protein degradation, interaction, and transport.

### 3.3. Stress and Defense

On the basis of proteomic profiles, we found six differentially expressed proteins responding to stress and defense, 2 of which were upregulated and 4 of which were downregulated in the H group. The upregulated expression of allene oxide cyclase (A0A103SI29, EC 5.3.99.6, AOC) was more obvious. AOC catalyzes the stereospecific cyclization of an unstable allene oxide to (9S,13S)-12-oxo-(10,15Z)-phytodienoic acid, the ultimate precursor of jasmonic acid (JA) [29]. There are more reports that JA is a natural hormone regulator that participates in development, pathogen attack and responses against wounding [30]. JA has been demonstrated to be an effective inducer of secondary metabolites in plant cells. For example, it has been reported that JA can significantly promote the synthesis of a triterpenoid saponin [31]. JA signal can increase the activity of HMG-CoA reductase (HMGR) [32], which is a previously reported enzyme in dipsacus saponin VI biosynthesis pathway (Figure 7). The formation of a triterpenoid saponin in *D. asperoides*, dipsacus saponin VI, may also be related to JA. The downregulated protein was primarily involved in the antioxidant process of cells; therefore, the antioxidant ability of *D. asperoides* in group H was weaker than that in groups Y, G, and S.

### 3.4. Secondary Metabolism

We found that two downregulated differentially expressed proteins were involved in the secondary metabolism of alkaloids and triterpene saponins in the H group. Putative tropinone reductase 1 (A0A251RSK1, EC 1.1.1.206, TRI), which has been considered to be an important regulatory target in the tropane alkaloids biosynthetic pathway [33]. Tropane alkaloids are a class of important secondary metabolites produced by various plant species and play an important role in plant defense against herbivore insects [34]. Tropane alkaloids may be among the active ingredients in *D. asperoides* to exert their pharmacological effects. However, there are no reports on tropane alkaloids in *D. asperoides* at present. In the future, our research group will conduct further studies on tropane alkaloids in *D. asperoides*. Isopentyl diphosphate isomerase 2 (A0A059PYD4, EC 5.3.3.2, IDI-2) is an enzyme required for the synthesis of isoprenoid metabolites via the mevalonic acid (MVA) pathway [35]. IDI-2 catalyzes the interconversion of isopentenyl diphosphate (IPP) and dimethylallyl diphosphate (DMAPP) in the isoprenoid biosynthetic pathway [36] (Figure 7). Triterpenoid saponin in plants is composed of isopentenyl diphosphate (IPP) and C5 isoprene unites, which are primarily supplied from the cytosolic MVA pathway and play a major role in the production of triterpenoidal sapogenin backbones [37]. At present, there is no detailed report on the synthesis of triterpenoid saponins (dipsacus saponin VI) in *D. asperoides*. We speculate that IDI-2 plays a key role in the synthesis of dipsacus saponin VI in *D. asperoides*.

### 3.5. Nucleic Acid Metabolism

In our proteomic profiles, we found two upregulated differentially expressed proteins involved in nucleic acid metabolism in the H group. Argonaute/Dicer protein (A0A103Y950, AGO) is the direct binding partner of small RNAs and plays a role in cell transcription, alternative splicing, and DNA repair [38]. Due to environmental reactions, the specific AGO proteins have unique biochemical activities and indicate the diversity of function and structure, as well [39]. In this study, the environmental factors of *D. asperoides* growth in different habitats are different. Perhaps AGO protein may play a role in the response of *D. asperoides* to environmental stress.

### 3.6. Amino Metabolism and Cell Wall Synthesis

We found that four differentially expressed proteins were involved in amino metabolism and cell wall metabolism in the H group, respectively. These differential proteins are primarily involved in the synthesis of some precursors in amino metabolism and cell wall synthesis.

### 3.7. Relationship Between Proteomic and Metabolomic Analysis in Different Habitats of D. asperoides

To better reveal the differential metabolism of *D. asperoides* from different habitats, the following results were obtained through the analysis of differential proteins involved in metabolic pathways. (1) Through the analysis of the differential proteins, we found that the protein, energy, and carbon metabolism of *D. asperoides* from Hubei province were robust. (2) Eight proteins, fructose-bisphosphate aldolase, glyceraldehyde-3-phosphate dehydrogenase, putative eukaryotic aspartyl protease family protein, elongation factor 1-alpha, heat shock protein 70, putative heat shock protein 81-2, putative tropinone reductase 1, and argonaute/dicer protein, were found in *D. asperoides* in response to environmental stress from different habitats, which could provide basic data for future selection of new *D. asperoides* varieties of good quality using molecular plant breeding technology. (3) Dipsacus saponin VI is the main active ingredient of *D. asperoides*. At present, the pathway of synthesis of dipsacus saponin VI has not been determined. We found that three proteins, UTP-glucose-1-phosphate uridylyltransferase, allene oxide cyclase, and isopentyl diphosphate isomerase 2, may be the key proteins involved in dipsacus saponin VI synthesis. According to previous reports, we have inferred the biosynthesis pathway of dipsacus saponin VI (Figure 7). Our findings of candidate enzymes involved in dipsacus saponin VI biosynthes provide novel information for us to understand the synthesis pathway of triterpenoid saponins in the future.

## 4. Materials and Methods

### 4.1. Sample Collection

All samples were collected in early October 2018, and the samples had been planted for three years when harvested. The roots of *D. asperoides* were sampled from Hefeng city, Hubei province (29°82′58″ N, 110°58′22″ E, H group); Xichang city, Sichuan province (28°4′4″ N, 102°8′36″ E, S group); Xifeng city, Guizhou province (27°10′73″ N, 106°74′38″ E, G group); and Jianchuan city, Yunnan province (26°31′91″ N, 99°85′24″ E, Y group), China. Twenty-five well-growing roots of *D. asperoides* were collected from each of the four places, and their quality accorded with the requirements of Chinese 2015 Pharmacopoeia. The sample was rinsed with water, the surface water was absorbed by absorbent paper, and the sample was immediately frozen with liquid nitrogen and stored at −80 °C.

### 4.2. Determination of Dipsacus Saponin VI

Dipsacus saponin VI was determined according to the method of Chinese Pharmacopoeia 2015. *D. asperoides* roots were dried and ground into powder. Then, 0.5 g powder was and soaked in 25 mL methanol, ultrasonicated for 30 min (power, 100 W; frequency, 40 kHz), allowed to cool, weighed, and membrane-filtered. Five milliliters of filtrate was accurately measured, put it into a 50-mL measuring bottle, mobile phase was added to dilute it to the scale, and it was shaken well. The diluent (20 μL) was analyzed for dipsacus saponin VI on a C18 symmetry column (4.6 × 250 mm, 5 μm) on a Waters HPLC system, with the following chromatography parameters: detection wavelength, 212 nm; mobile phase, acetonitrile-water (30:70); flow rate, 1.0 mL/min; and theoretical plate number, ≥3000. Standard dipsacus saponin VI (purity, 91.3%; JY8R-BINA2) was obtained from the China Food and Drug Certification Research Institute (Beijing, China). Six biological repeats were made in the determination of dipsacus saponin VI content in samples from each habitat.

### 4.3. Protein Extraction, Digestion, and iTRAQ Labeling

The modified Tris-HCl method was used to extract protein [18]. Next, 4.0 g of *D. asperoides* roots frozen at −80 °C was ground to a powder in liquid nitrogen. Then, the powder was placed in a centrifuge tube, and 10 mL of protein extraction buffer (65 mmol/L Tris-HCl pH 6.8, 10% glycerol (*v*/*v*), 5% β-mercaptoethanol (*v*/*v*), and 0.5% SDS (*w*/*v*) were added. The centrifuge tube was placed in a 4 °C temperature-controlled shaker and shaken for 1 h. Next, samples were subjected to centrifugation at 4 °C and 15,000× *g* for 15 min. After that step, the protein supernatant was taken, 3 volumes of a pre-cooled 10% TCA acetone solution at −20 °C was added, and the mixture was fully blended and placed in a refrigerator at −20 °C for an hour to precipitate the protein. The supernatant was discarded after centrifugation for 15,000× *g* for 15 min at 4 °C, and the pellet was washed twice with equal amounts of precooled acetone (containing 0.07% β-mercaptoethanol (*v*/*v*) and 80% precooled acetone (*v*/*v*). After centrifugation at 15,000× *g* for 15 min at 4 °C, the pellet was vacuum-dried and stored in a refrigerator at −80 °C. Bradford’s method was used for protein quantification.

For each sample, 100 µg protein in TEAB buffer was incubated in a sealed tube for 24 h with 3.3 UG trypsin (1 ug/mL) (Promega, Madison, WI, USA) at 37 °C. The tryptic peptides were lyophilized and dissolved in 50% TEAB buffer. According to the manufacturer’s instructions (AB Sciex lnc., Redwood City, MA, USA), samples were labeled with iTRAQ reagent 8-Plexkit. The samples from Hefeng (H group) were labeled with iTRAQ tag 117, those from Xifeng (G group) were labeled with tag 118, those from Jianchuan (Y group) were labeled with tag 119, and those from Xichang (S group) were labeled with tag 121. The labeled samples were incubated at room temperature for 2 h, and then the mixture of peptides was mixed and dried in vacuum.

### 4.4. Strong Cation Exchange

The labeled samples were alkalinized and fractionated using a strong cation-exchange chromatography HPLC system (Agilent 1100, Santa. Clara, CA, USA) connected to the SCX column (Luna 5u column, 4.6 × 250 mm, 5 μm, 100 Å; Phenomenex, Torrance, CA, USA). The retained peptides were eluted with buffer A (10 mM KH_2_PO_4_ in 25% acetonitrile aqueous solution, acidified with H_3_PO_4_ to a pH of 3.0) and buffer B, which consisted of buffer A and 2 M KCl. The fractions were collected in 1.5 mL microtubules with a flow rate of 1 mL/min. The following chromatographic gradients were used: 0–25 min 100% buffer A; 0–10 min 5% buffer B; 10–40 min 5–30% buffer B; 40–45 min 30–60% buffer B, 45–55 min 60–80% buffer B; and 55–65 min decreasing to 5% buffer B. Fractions were collected every minute after 26 min. The elution fractions were dried in a vacuum concentrator, and each fraction was dissolved in 0.1% formic acid solution before reversed-phase nano-LC-tandem mass spectrometry (LC-MS/MS).

### 4.5. NanoLC–MS/MS Analysis by Q Exactive

The peptides were dissolved in 50 μL mobile phase A (0.1% formic acid) and loaded onto an Acclaim PePmap C18-reversed phase column (75 μm × 2 cm, 3 μm, 100 Ǻ Thermo Scientific). The peptides were isolated by reversed phase C18 column (75 μm × 10 cm, 5 μm, 300 Ǻ, Agela Technologies, New York, NY, USA), which was mounted on a Dionex ultimate 3000 nano LC system. Peptide elution was performed with the following gradient: 0–6 min 5% buffer B; 6–6.5 min 10% buffer B; 6.5–45 min 10–24% buffer B; 45–51 min 24–40% buffer B, 51–54 min 40–80% buffer B; 54–59 min 80% buffer B; 59–59.9 min 5% buffer B; 59.9–65 min 5% buffer B. This method was used in combination with a Q Exactive mass spectrometer (Thermo Fisher Scientific, Waltham, MA, USA) at a flow rate of 300 nL/min. The eluates went directly to Q-Exactive MS, set to cation mode, and relied on data with full MS scanning at 350–2000 *m*/*z*, 70,000 full scanning resolution, and MS/MS scanning resolution at 17,500. For MS/MS scans, the minimum signal threshold was 1 × 10^5^, and the isolation width was 2 Da. To evaluate the performance of this mass spectrometry on iTRAQ-labeled samples, two MS/MS acquisition modes and higher collision energy dissociation (HCD) were used. To optimize the MS/MS acquisition efficiency of HCD, the normalized collision energy (NCE) was systemically checked 28 times, stepped 20%.

### 4.6. Protein Identification

The original files were converted to MASCOT generic format (.mgf) files by default settings of Proteome Discoverer 1.4 (Thermo Fisher Scientific) for deep proteome analysis. Protein Pilot 5.0 (AB Sciex, Foster City, CA, USA) was used for protein quantitation and deep proteome analysis with .mgf files as input.

In detail, these parameters were as follows: “Thorough ID” mode with 95% confidence level, iTRAQ peptide labeling, trypsin digestion, and Cys oxidation by methyl methanethiosulfonate (MMTS). To increase confidence level, proteins with an iTRAQ ratio greater than 20 or less than 0.05 were not considered to be quantified, and only proteins that are reasonable ratios across all channels were recognized to be quantitative.

### 4.7. Bioinformatics Analysis

Genetic function clustering GO analysis of differential proteins was performed using the QuickGO (https://www.ebi.ac.uk/QuickGO/) annotation tool maintained by the European Bioinformatics Institute (EMBL-EBI). The KEGG pathway database was used to analyze the metabolic pathways involved in differential proteins, and STRING was used to analyze the network pathways of differential proteins.

### 4.8. Quantitative PCR Detection

Total RNA was isolated from frozen samples using TaKaRa MiniBEST Plant RNA Extraction Kit (TaKaRa, Dalian, China) according to the manufacturer’s instructions. RNA purification and complementary DNA (cDNA) synthesis were conducted with the PrimeScript^®^ RT Reagent Kit with gDNA Eraser (TaKaRa, Dalian, China). Quantitative real-time PCR (qRT-PCR) was performed on a CFX96 Real-Time PCR instrument (Bio-Rad, Hercules, CA, USA) using TB Green^®^ *Premix Ex Taq*™ for detection. The qRT-PCR mixture contained 12.5 μL 2× TB Green *Premix Ex Taq* II (TaKaRa. Bio, Siga, Japan), 2 μL of tenfold-diluted cDNA, 1 μL of 100 nM of each sense and antisense primer, and 8.5 μL of double-distilled water. The *18sRNA* gene was used as the internal control. Primer sequences for *18sRNA*, *FAB*, *galF*, *AOC*, *IDI2*, *UGDH*, and *CP* are listed in Table 3.

The PCR conditions were as follows: 95 °C for 30 s, 39 cycles at 95 °C for 5 s, 60 °C for 30 s. The relative messenger RNA (mRNA) expression of the target gene was calculated using the CT method. All experiments were performed in triplicate.

## 5. Conclusions

We found that the content of dipsacus saponin VI in Hubei was the highest by HPLC. In an initial attempt to elucidate the bioinformatics differences of *D. asperoides* from different habitats using the proteomics technology, we obtained 2149 proteins, and 202 differentially expressed proteins were screened. Through the integrated analysis of GO/KEGG/STRING of 202 differentially expressed proteins, 42 significantly differentially expressed proteins were screened out. Through in-depth analysis of differentially expressed proteins, we found that the protein metabolism and energy and carbohydrate metabolism of *D. asperoides* cells from Hubei Province were strong, but their antioxidant capacity was weak. Eight proteins, fructose-bisphosphate aldolase, glyceraldehyde-3-phosphate dehydrogenase, putative eukaryotic aspartyl protease family protein, elongation factor 1-alpha, heat shock protein 70, putative heat shock protein 81-2, putative tropinone reductase 1, and argonaute/dicer protein, were found in *D. asperoides* in response to environmental stress from different habitats, which could provide basic data for future selection of new *D. asperoides* varieties of good quality using molecular plant breeding technology. UTP-glucose-1-phosphate uridylyltransferase, allene oxide cyclase and isopentyl diphosphate isomerase 2 identified in this study could be used as key proteins involved in dipsacus asperoides VI synthesis. Our findings of candidate enzymes involved in dipsacus saponin VI biosynthes provide novel information for us to understand the synthesis pathway of triterpenoid saponins in the future. The results of this study may provide the basic information for exploring the cause of differences in secondary metabolites in different habitats of *D. asperoides* and the protein mechanism governing differences in quality.

## Figures and Tables

**Table 1:** Pathway enrichment analysis of differential expression of proteins.

Pathway	Number of Proteins	Pathway
Ribosome	32	map03010
Carbon metabolism	18	map01200
Biosynthesis of amino acids	12	map01230
Carbon fixation in photosynthetic organisms	10	map00710
Starch and sucrose metabolism	10	map00500
Glycolysis/Gluconeogenesis	9	map00010
Phenylpropanoid biosynthesis	9	map00940
Cysteine and methionine metabolism	6	map00270
MAPK signaling pathway-plant	5	map04016
Glyoxylate and dicarboxylate metabolism	4	map00630
Cyanoamino acid metabolism	4	map00460
Glutathione metabolism	4	map00480
Ubiquitin mediated proteolysis	3	map04120
Protein processing in endoplasmic reticulum	3	map04141
Plant-pathogen interaction	3	map04626

**Table 2:** Significantly differentially expressed proteins in *D. asperoides* root proteins from different producing areas.

Accession	Description	Score	Peptides	MW [kDa]	Calc. pI	117:118	117:119	117:121
**Energy and Carbo hydrate Metabolism**								
A0A161YFV4	Fructose-bisphosphate aldolase	287.2	10	38.3	6.77	2.244	1.905	1.791
A0A103Y5R9	UTP-glucose-1-phosphate uridylyltransferase	278.14	8	52.8	7.17	1.468	1.926	2.029
A0A251SNE7	Tubulin beta chain	58.38	8	50.5	4.86	1.428	1.9733	1.295
N0BKE3	Glyceraldehyde-3-phosphate dehydrogenase	993.14	12	37	7.94	2.225	1.834	3.063
A0A251UDE6	Putative glycosyl hydrolase family protein	23.63	2	75.5	8.78	1.354	1.467	1.286
A0A251UAJ0	Putative alcohol dehydrogenase superfamily, zinc-type	93.15	4	38.7	6.65	1.336	1.263	1.365
A0A161WPJ1	Dihydrolipoyl dehydrogenase	6.31	1	60.3	7.24	1.322	1.976	1.349
A0A251S8R8	Putative class II aaRS and biotin synthetases superfamily protein	15.94	3	54.7	5.86	1.277	1.286	1.205
A0A251V9F9	Putative glyceraldehyde/Erythrose phosphate dehydrogenase family	7.03	2	26.4	6	1.276	1.932	1.134
A0A2J6M8S6	Pyruvate dehydrogenase E1 component subunit beta	13.9	2	40.6	5.6	1.248	1.247	1.147
A0A103YI90	AAA+ ATPase domain-containing protein	109.03	5	163.7	6.49	0.735	0.924	0.840
A0A251UWR3	Mitochondrial pyruvate carrier	16.51	1	5.9	8.22	0.756	0.885	0.816
**Protein Metabolism**								
A0A251TTV4	Putative eukaryotic aspartyl protease family protein	42.48	2	48.4	7.49	2.0198	1.344	1.627
P26791	Heat shock protein 70	215.19	10	72	5.25	2.013	1.817	1.710
A0A251ST32	Putative heat shock protein 81-2	310.6	19	79.9	5.03	1.618	1.882	1.206
A0A251S512	40S ribosomal protein S24	19.77	2	14.1	10.54	1.615	1.297	1.462
A0A124SB86	60S ribosomal protein L13	21.85	4	23.6	11.17	1.465	1.390	1.209
A0A2J6JLK0	Elongation factor 1-alpha	405.87	14	49.3	9.07	1.338	1.417	1.657
A0A166JH20	Peptidyl-prolyl cis-trans isomerase	2.28	2	18.7	7.81	1.315	1.221	1.344
A0A165YIX7	Proteasome subunit alpha type	32.64	2	27.4	6.3	1.312	1.424	1.989
A0A251VP43	Putative nascent polypeptide-associated complex subunit alpha-like protein 3	78.95	3	21.9	4.55	1.3008	1.218	1.288
A0A124SH07	Chaperonin 21, chloroplast	1.82	1	26.4	8.05	1.3045	1.364	1.278
A0A251T242	Argonaute/Dicer protein	23.03	3	17.3	10.29	1.283	1.441	1.285
A0A166BV50	40S ribosomal protein S6	15.74	4	28.2	10.67	1.241	1.340	1.333
A0A251V9W6	Putative 14-3-3 domain-containing protein	60.62	5	29.3	4.83	0.6296	0.753	0.502
A0A161Y785	Carboxypeptidase	11.44	2	50.2	7.37	0.7179	0.668	0.535
A0A103YD00	Ubiquitin-conjugating enzyme/RWD-like protein	2.69	1	19.4	7.88	0.758	0.601	0.779
A0A118JY32	Protein transport protein Sec61 subunit beta	3.8	1	8.5	10.96	0.785	0.508	0.746
**Amino Metabolism**								
A0A251SNP4	Putative 5-methyltetrahydropteroyltriglutamate homocysteine methyltransferase	707.58	14	92.5	6.76	1.309	1.944	1.220
A0A0S2C3Q5	Phospho-2-dehydro-3-deoxyheptonate aldolase	27.93	2	25.6	6.9	0.6488	0.614	0.572
**Stress and Defense**								
A0A103SI29	Allene oxide cyclase	9.34	1	17.1	9.54	1.8996	1.377	1.465
A0A251UFC8	Phosphoinositide phospholipase C	9.63	2	20.2	5.72	1.2614	1.2095	1.431
A0A103Y696	Peroxidase	6.64	1	34.2	7.83	0.702	0.456	0.573
A0A251VSF3	Putative berberine/berberine-like, FAD-binding, type 2	23.91	3	61	4.97	0.714	0.703	0.518
Q8RVT5	Acyl-CoA-binding protein	15.33	1	9.9	5.5	0.777	0.977	0.834
Q93YG5	Superoxide dismutase (Fragment)	8.53	2	15.9	6.27	0.794	0.602	0.546
**Nucleic Acid Metabolism**								
A0A103Y950	Argonaute/Dicer protein	37.43	7	120.9	9.39	1.2796	1.230	1.062
A0A251SDZ7	Putative DNA/RNA-binding protein Alba-like protein	5.63	1	25.2	10.24	1.255	1.444	1.267
**Cell Wall Synthesis**								
A0A103Y4W7	UDP-glucose 6-dehydrogenase	51.35	6	53.1	5.85	1.224	1.399	1.138
A0A103XC31	Glucose/ribitol dehydrogenase	3.42	1	34.3	6.8	0.607	0.975	0.890
**Secondary Metabolism**								
A0A251RSK1	Putative tropinone reductase 1	20.44	2	31.8	0.709	0.767	0.713	0.671
A0A059PYD4	Isopentyl diphosphate isomerase 2	26.67	4	32.5	5.31	0.782	0.880	0.720

**Table 3:** Genes and primers used for quantitative PCR.

Gene	Protein	Forward/Reverse Primer Sequence (5′→3′)
*FBA*	Fructose-bisphosphate aldolase	Forward primer AGTACTGCTGCTGGAAAACCT Reverse primer CATCGGTACCAGCAAGCTCA
*galF*	UTP-glucose-1-phosphate uridylyltransferase	Forward primer GGCTGCTGCTGATACCGA Reverse primer GCATCCCATTGTTGTCCC
*AOC*	Allene oxide cyclase	Forward primer TCTATGTTATCTACGGAATGG Reverse primer AACCGAAAGGTAGGCATC
*IDI2*	Isopentyl diphosphate isomerase 2	Forward primer TCTATGTTATCTACGGAATGG Reverse primer GAGAAAGACGAGCGAGGT
*UGDH*	UDP-glucose 6-dehydrogenase	Forward primer ACATCATCACGACCAATCT Reverse primer GTCCTTACCAACGGCATA
*CP*	Carboxypeptidase	Forward primer CTAAAGTGGGAAGGCATAA Reverse primer TACAGGGCTGATCTACGG
*18sRNA*		Forward primer AGCAGATTGACCAGCGAACA Reverse primer CAGAAAGGAGCACCACCC

## Supplementary Materials

The following are available online. Table S1: Raw data of identification and analysis of the *Dipsacus asperoides* proteome; Table S2: Information on 202 screened differentially expressed proteins. Table S3: String protein annotations.
